# Functional and Effective Connectivity Characteristics of the Dorsolateral Prefrontal Cortex in Drug-Naïve Patients with Generalized Anxiety Disorder

**DOI:** 10.31083/AP51187

**Published:** 2026-09-03

**Authors:** Lijuan Yang, Fang He, Danfeng Yuan, Ting Yang, Liyang Zhang, Yu Chen, Jia Luo, Ying Li, Xiangyun Yang, Zhanjiang Li

**Affiliations:** ^1^Beijing Key Laboratory of Mental Disorders, National Clinical Research Center for Mental Disorders & National Center for Mental Disorders, Beijing Anding Hospital, Capital Medical University, 100088 Beijing, China; ^2^Advanced Innovation Center for Human Brain Protection, Capital Medical University, 100069 Beijing, China

**Keywords:** generalized anxiety disorder, functional magnetic resonance imaging, functional connectivity, effective connectivity

## Abstract

**Background:**

The dorsolateral prefrontal cortex (dLPFC) is a key brain region involved in cognitive control and emotional regulation and has been implicated in the pathophysiology of generalized anxiety disorder (GAD). This study used resting-state functional magnetic resonance imaging (rs-fMRI) to investigate functional connectivity (FC) and effective connectivity (EC) patterns of the bilateral dLPFC in drug-naïve patients with GAD. In addition, the relationships between abnormal connectivity patterns, anxiety symptoms, and attentional bias (AB) were examined.

**Methods:**

A total of 40 patients with GAD and 40 healthy controls (HCs) were recruited. Clinical symptoms in the GAD group were assessed using the Hamilton Anxiety Rating Scale (HAMA), the 17-item Hamilton Depression Rating Scale (HAMD-17), and the Generalized Anxiety Disorder-7 (GAD-7) scale. Attentional bias toward negative stimuli was evaluated using the dot-probe task and the emotional Stroop task. Independent-samples *t*-tests were performed to compare whole-brain voxel-wise FC and EC differences based on bilateral dLPFC regions of interest (ROIs) between the two groups. Partial correlation analyses, controlling for the duration of the current episode, were conducted to examine associations between aberrant FC/EC values and clinical symptoms or attentional bias in patients with GAD.

**Results:**

Compared with healthy controls, patients with GAD exhibited increased FC between the dLPFC and the medial superior frontal gyrus, inferior parietal gyrus, and left precentral gyrus. In addition, patients with GAD showed reduced directional (Granger-causal) connectivity from the dLPFC to the caudate nucleus, thalamus, and BA37, as well as from the left postcentral gyrus to the dLPFC (voxel-level *p* < 0.01; cluster-level Gaussian random field [GRF]-corrected *p* < 0.05). Correlation analyses revealed that dLPFC-based FC and EC values were significantly associated with anxiety symptom severity and attentional bias to varying degrees (r = −0.515 to 0.584, *p* < 0.05).

**Conclusions:**

This study identified widespread abnormalities in resting-state FC and EC involving the bilateral dLPFC in patients with GAD. Aberrant FC and EC patterns were closely associated with anxiety symptoms and attentional bias, further supporting the anxiety network hypothesis and highlighting the central role of the dLPFC within anxiety-related neural circuits. These findings provide preliminary neuroimaging evidence for the cognitive model of GAD and suggest that attentional bias toward threat-related stimuli may play a critical role in the pathogenesis of the disorder.

## Main Points

1. Patients with generalized anxiety disorder (GAD) tend to focus on worry-related information and are inclined to interpret ambiguous information as threatening. This attentional bias toward negative information is considered a key pathological mechanism in the development and maintenance of GAD. Its neural basis may be associated with a reduced regulatory capacity of the dorsolateral prefrontal cortex (dLPFC) over subcortical brain regions.

2. Patients with GAD exhibit abnormalities in both functional connectivity (FC) and effective connectivity (EC) of the bilateral dLPFC. In addition to alterations in the classic prefrontal-limbic circuit connections, patients also display abnormal connectivity patterns involving brain regions related to the sensorimotor network and the basal ganglia network. These findings further enrich and expand the neuropathological model of GAD.

3. Abnormalities in dLPFC-related functional and effective connectivity may play a significant role in the selective attention to threat-related stimuli observed in patients with GAD. Therefore, neuromodulatory interventions targeting the dLPFC may represent a potential therapeutic target, warranting further investigation in controlled clinical trials.

## 1. Introduction

Generalized anxiety disorder (GAD) is the most common type of anxiety disorders, characterized by chronic, excessive and uncontrollable worry about a variety of topics [1]. The prevalence of GAD in the adult population is approximately 2%, with a lifetime prevalence of around 4.7% [2]. GAD frequently co-occurs with other anxiety and mood disorders and has the lowest remission rate after treatment compared to other anxiety disorders [3,4]. Neuroimaging studies, by identifying structural and functional changes in the brains of individuals with GAD, may contribute to a better understanding of its neurobiological mechanisms. However, research in this domain remains relatively limited for GAD and findings to date have been inconsistent [5].

According to recent reviews and cognitive models of anxiety, patients with GAD maintain a high level of vigilance towards threatening stimuli and exhibit a tendency to interpret ambiguous information as threatening [6]. This attentional bias (AB) is considered a stable trait-like characteristic and plays a key role in the development and maintenance of GAD [7]. Research indicated that the degree of AB was closely associated with the level of anxiety [8]. Furthermore, cognitive bias modification studies provided additional evidence supporting the causal role of threat-related AB in the core psychopathology of GAD [9]. Neuroimaging studies have indicated that AB toward threat-related information is closely associated with disrupted functional balance between the prefrontal cortex (PFC) and the limbic system [10,11]. Specifically, the limbic system, particularly the amygdala, is responsible for monitoring potential threat-related information in the environment, whereas the PFC, especially regions such as the dorsolateral PFC (dLPFC), exerts inhibitory control over attention by suppressing task-irrelevant distractors to maintain top-down attentional regulation [12,13]. Bishop and colleagues proposed an integrative neurocognitive model of AB in anxiety, suggesting that the dLPFC exerts regulatory control over the amygdala. When this dLPFC-mediated control over the amygdala fails, patients exhibit heightened AB [14].

Moreover, heightened AB toward negative information has been consistently reported across anxiety-related conditions and is considered a transdiagnostic vulnerability marker [15]. Neuroimaging evidence further suggests that individual differences in AB are closely linked to prefrontal regulatory capacity over limbic and sensory regions, providing a mechanistic framework for investigating dLPFC-based connectivity alterations in GAD [16]. Notably, difficulties in identifying and regulating internal affective states—such as alexithymic features—have been linked to heightened AB and altered frontolimbic processing in anxiety populations [17], supporting the view that AB represents a cognitive–affective marker embedded within broader emotion regulation circuitry.

The dLPFC, serving as a key neural substrate for cognitive operations such as working memory and attention, as well as a critical region for regulating emotional responses, has become a focal area in research on the neurobiological basis of cognitive control and emotion regulation dysfunction in GAD [18]. Price et al. [19] employed the Stroop test combined with fMRI to investigate the mechanisms of abnormal selective attention in GAD patients and revealed that participants’ attention was easily disrupted by negative information, accompanied by reduced functional activity in the PFC. A proton magnetic resonance spectroscopy study demonstrated that GAD was associated with a low level of choline/N-Acetylaspartate in the dLPFC, which is closely related to symptom severity and cognitive dysfunction [20]. A tDCS study targeting individuals with trait anxiety found that stimulation of the dLPFC could reduce amygdala threat responses while enhancing activity in key nodes of both dorsal and ventral attention control networks, which offered the first causal evidence for prefrontal regulation of amygdala function in attentional capture by threat in individuals with high trait anxiety [21].

Although most studies consistently report abnormalities in the prefrontal-limbic circuit in GAD patients, the specific localization of affected brain regions varies due to differences in experimental paradigms, medication history, comorbidities, and other factors [5,10]. A recent meta-analysis further indicates that GAD-related functional areas have expanded beyond the classic regions—such as the dLPFC, anterior cingulate cortex (ACC), amygdala, and hippocampus—to include broader brain networks [22]. This suggests that the neural basis of GAD likely involves whole-brain structural alterations rather than being confined to specific anatomical structures.

To address this, the present study recruited medication-naïve GAD patients and healthy controls (HCs), collecting rs-fMRI data and clinical measures. Additionally, the dot-probe task and emotional Stroop paradigm were employed to assess AB toward threat-related stimuli in patients. The study aims to: (1) identify altered dLPFC-centered FC networks in GAD; (2) apply Granger causality analysis (GCA) to characterize abnormal directional information flow between the dLPFC and other brain regions; (3) investigate the relationship between FC/EC strength, directional connectivity patterns, and clinical anxiety severity as well as AB indices.

## 2. Materials and Methods

### 2.1 Participants

The study was conducted at Beijing Anding Hospital, with a period of active recruitment from April 2023 to May 2025. Forty patients with GAD (26 females and 14 males) were recruited from Beijing Anding Hospital Outpatient Department. All patients were diagnosed by psychiatrists according to the criteria of the Diagnostic and Statistical Manual of Mental Disorders, Fifth Edition (DSM-5) using a mini-international neuropsychiatric interview, 7.0 version (M.I.N.I.7.0). 40 HCs (27 females and 13 males) were recruited from the local community through a recruitment advertisement. All participants were right-handed (based on the Edinburgh Handedness Inventory). The GAD inclusion criteria were: (1) patients who met the diagnostic criteria for GAD according to DSM-5; (2) score ≥14 on the Hamilton Anxiety Scale (HAMA), and <17 on the 17-item Hamilton Depression Rating Scale (HAMD-17); (3) patients aged between 18 and 60 years and had an education level of junior high school or above; (4) patients with no history of neurological illness or other major physical illness, other psychiatric disorders, psychoactive substance use, alcohol dependence, and/or alcohol abuse; (5) patients who had no history of exposure to anti-depressants, psychotherapy, or physical therapy; (6) no contraindications to MRI scanning. HCs were enrolled if they were aged between 18 and 60 years and had an education level of junior high school or above. The exclusion criteria for the HCs included a neurological illness, major physical diseases, Axis I psychiatric disorders, or a positive family history of major psychiatric disorders.

### 2.2 GAD Measurements and Evaluation

#### 2.2.1 Hamilton Anxiety Scale (HAMA)

The HAMA is the most widely used assessment tool for anxiety disorders in psychiatric clinical practice and research [23]. It is a 14-item clinician-administered questionnaire divided into two dimensions: “psychic anxiety” and “somatic anxiety”, rated on a five-point scale (0–4), with total scores ranging from 0 to 56.

#### 2.2.2 17-item Hamilton Depression Rating Scale (HAMD-17)

The HAMD-17, developed by Max Hamilton in 1960, is the most widely used depression assessment instrument in psychiatric clinical practice and research [24]. This 17-item clinician-administered scale evaluates core depressive symptoms including depressed mood, sleep disturbances, and somatic symptoms. It employs a 0–4 five-point scoring system for most items (with a minority of items using 0–2 three-point scoring), demonstrating high sensitivity to changes in depressive symptomatology.

#### 2.2.3 Generalized Anxiety Disorder-7 (GAD-7)

The GAD-7 is a concise self-rating scale for anxiety symptoms developed by Spitzer’s team based on the diagnostic criteria of DSM-4 [25]. The GAD-7 primarily assesses a patient’s anxiety symptoms over the past two weeks, using a scale from 0 (absence) to 3 (extreme), with a total score range of 0–21. When the cutoff score is set at 10, the scale achieves optimal sensitivity and specificity at 89% and 82%, respectively. The GAD-7 can not only be used to screen for GAD but also to evaluate the severity of the condition. Scores of 5, 10, and 15 correspond to mild, moderate, and severe anxiety disorders, respectively.

### 2.3 Attentional Bias Evaluation in GAD

#### 2.3.1 Dot-Probe Task

This task is designed to measure participants’ AB toward negative images [26]. The procedure consists of 10 practice trials followed by 96 experimental trials. Each trial begins with a central fixation cross displayed on the screen for 500 milliseconds. After the cross disappears, two images (one threatening and one neutral) are presented for 500 milliseconds. Following the disappearance of the images, a probe (the letter “X”) immediately replaces one of the images. Participants are required to press the corresponding button (F key for the left side, J key for the right side) to indicate the probe’s location. The stimuli include 24 distinct image pairs, each featuring a neutral-fear image combination. The images are randomly selected from the Chinese Affective Picture System [26], with each pair appearing 4 times. The attentional bias score is calculated by subtracting the reaction time for probes in the negative image location from the reaction time for probes in the neutral image location (in milliseconds). A positive value indicates an attentional bias toward threatening images, while a negative value suggests attentional avoidance of threatening images.

#### 2.3.2 Emotional Stroop Task

This task is used to measure participants’ attentional bias toward negative words [27]. Participants are required to identify the color of the words (red, yellow, or green) while ignoring their meanings. When a word appears on the screen, they must press one of three designated keys corresponding to the word’s color. All words are presented once in each of the three possible colors to avoid biases related to color effects or button positions. The word set includes neutral, negative, and positive terms, selected from the Modern Chinese Affective Word System based on their arousal, valence, and dominance ratings [28]. The task follows a three-block design, with each stimulus displayed for 1000 milliseconds followed by a 500-millisecond blank screen (white). Each block consists of 96 trials (32 words per emotional category). The attentional bias score is calculated by subtracting the reaction time for neutral words from that for negative words. A positive value indicates delayed color-naming for negative words (suggesting attentional bias), while a negative value suggests faster processing of negative words.

### 2.4 fMRI Data Acquisition and Processing

All participants underwent MRI scanning at the Radiology Department of Beijing Anding Hospital using a Siemens 3.0 T MAGNETOM Prisma MRI scanner (Siemens Medical Solutions, Erlangen, Germany) equipped with a 64-channel phased-array head coil. Participants were instructed to remain still, stay relaxed but awake, and avoid focusing on any specific thoughts during the scan. Foam padding was provided to minimize head movement, and earplugs were used to reduce scanner noise. Following anatomical localization, resting-state functional images were acquired using an echo-planar imaging (EPI) sequence, yielding 200 volumes of axial images. Key acquisition parameters included: Slices = 33, Repetition time (TR) = 2000 ms, Echo time (TE) = 30 ms, Flip angle = 90°, Field of view (FOV) = 200 × 200 mm^2^, Matrix size = 64 × 64, Slice thickness = 3.5 mm, Voxel size = 3.13 × 3.13 × 4.20 mm^3^. High-resolution structural images were obtained using a T1-weighted magnetization-prepared rapid gradient-echo (MPRAGE) sequence with the following parameters: TR = 2530 ms, TE = 1.85 ms, Flip angle = 15°, FOV = 256 × 256 mm^2^, Slices = 192, Voxel size = 1 × 1 × 1 mm^3^.

### 2.5 fMRI Preprocessing and Analysis

Preprocessing of the fMRI images was performed using Data Processing Assistant for Resting-State fMRI (DPARSF) with the following steps: removing the first 10 volumes; slice timing correction; head motion realignment (subjects with head motion above 2 mm or 2° were excluded); co-registration and segmentation; regression for nuisance variables including linear trends, Friston’s 24 head motion parameters, white matter, cerebrospinal fluid, and global signal; spatial normalization to the Montreal Neurological Institute (MNI) template (resampled to 3 × 3 × 3 mm^3^) by DARTEL; spatial smoothing with a 4-mm FWHM Gaussian kernel; temporal bandpass filtering (0.01–0.1 Hz). A total of three GAD patients and one HC were excluded. The final sample was thus comprised of 37 GAD patients and 39 controls.

Voxel-wise FC analyses were conducted to explore the FC abnormalities using bilateral dLPFC as seeds. Pearson’s correlation coefficients of seeds with all remaining brain voxels were computed in DPARSF software after preprocessing. The correlation maps were then normalized using Fisher’s *r*-to-*z* transformation to *z* score FC maps to improve normality.

Voxel-wise EC analyses were conducted to explore the EC abnormalities using bilateral dLPFC as seeds. The seed-based EC analyses were performed using Resting-State fMRI Data Analysis Toolkit (REST). The bivariate coefficient-based GCA approach (based on an autoregressive model with time lag order of 1) was adopted to investigate the directional (Granger-causal) relationship between the selected seeds and all remaining brain voxels. It should be noted that Granger causality derived from resting-state fMRI reflects statistical predictability rather than true neural causality, given hemodynamic delays, the relatively slow sampling rate (TR = 2000 ms), and the influence of preprocessing steps on the BOLD signal.

The normalized *z* score EC maps were generated for the subsequent analysis.

### 2.6 Statistical Analyses

Statistical analyses were performed using SPSS 24.0 software (Armonk, NY, USA). Continuous variables with normal distribution were expressed as mean ± SD and compared using independent *t*-tests and non-normally distributed variables were presented as median (Q1, Q3) and analyzed using Mann–Whitney U test. Categorical variables were presented as number (percentage) and analyzed using chisquared test or Fisher’s exact test. A two-sample *t*-test was used to examine the FC differences between GAD and HC group with mean frame-wise displacement(FD) as covariates. Multiple comparisons correction using GRF correction (two-tailed voxel-level *p* < 0.01 and cluster-level *p* < 0.05) was conducted by DPABI software. A two-sample *t*-test was used to examine the EC differences between GAD and HC group with mean FD as covariates and multiple comparisons correction using GRF correction (two-tailed voxel-level *p* < 0.01 and cluster-level *p* < 0.05) was conducted in DPABI software.

Based on FC and EC comparisons between GAD patients and HCs, we identified brain regions with altered FC/EC patterns in GAD patients. The FC and EC values of these regions were then extracted for each GAD participants. Given that the variables involved in the correlation analysis were all continuous and normally distributed, we performed partial correlation analysis using the duration of the current episode as a covariate to examine the relationships between FC/EC strength, symptom severity, and AB values.

A post-hoc power analysis was conducted using G*Power 3.1 (Düsseldorf, North Rhine‑Westphalia, Germany) to evaluate the statistical power of the final sample. With 37 patients with GAD and 39 HCs, alpha = 0.05, and two-tailed testing, the achieved power was approximately 79.8% to detect a between-group effect size of Cohen’s d = 0.65. For patient-only correlation analyses in the GAD group, the achieved power was approximately 70.9% to detect a correlation of r = 0.40.

## 3. Results

### 3.1 Demographic and Clinical Data

The final sample included 37 patients with GAD and 39 HCs, and all GAD patients who were included in the analysis had not been treated with any medication or psychotherapy before the study. As shown in Table 1, the two groups were not statistically different for age (t = –0.024, *p* = 0.981), gender (χ^2^ = 0.018, *p* = 0.892), marriage status (χ^2^ = 1.547, *p* = 0.461) and years of education (t = –0.805, *p* = 0.424). There were significant differences HAMA, HAMD-17 total scores and GAD-7 scores between the two groups (t = 24.195, t = 23.372, t = 17.801; *p* < 0.001). The GAD group showed a significantly higher attentional bias score toward negative words measured by the emotional Stroop paradigm compared to the HCs (t = 2.394, *p* = 0.022), whereas the difference in attentional bias scores measured by the dot-probe paradigm did not reach statistical significance between the two groups (*p* > 0.05).

**Table 1.** **Demographic and clinical characteristics of patients with GAD and HCs**.

	GAD (n = 37)	HCs (n = 39)	Test value (F/χ^2^)	*p* value
Sex (male/female)	14/23	13/26	0.018	0.892
Age (year)	33.86 ± 6.89	33.90 ± 7.34	–0.024	0.981
Years of education	15.34 ± 2.35	15.74 ± 1.93	–0.805	0.424
% married	48.65	53.85	1.547	0.461
Duration of current episode (months), Median (interquartile range)	8.00 (6.00–12.00)	-	-	-
HAMA	17.09 ± 3.94	0.66 ± 0.80	24.195	<0.001
HAMD-17	11.28 ± 3.00	0.62 ± 0.87	23.372	<0.001
GAD-7	12.65 ± 3.83	0.74 ± 0.75	17.801	<0.001
AB measured by dot-probe task	1.41 ± 15.07	–2.38 ± 8.48	1.312	0.194
AB measured by emotional stroop task	3.18 ± 11.28	–8.36 ± 17.25	2.394	0.022

Notes: GAD, generalized anxiety disorder; HCs, healthy controls; HAMA, Hamilton Anxiety Scale; HAMD-17, Hamilton Depression Rating Scale; GAD-7, Generalized Anxiety Disorder-7; AB, attentional bias.

### 3.2 The rsFC and EC Analysis With Bilateral dLPFC as Seed Regions in GAD and HCs

We used bilateral dLPFC as seed regions and performed voxel-wise two-sample *t*-tests to examine between-group differences in rsFC and rsEC. The rsFC results were presented in Table 2 and Figs. 1,2. When seeding the left dLPFC, patients with GAD exhibited significantly increased FC with the right medial superior frontal gyrus (BA8). When seeding the right dLPFC, GAD patients showed significantly enhanced FC with left inferior parietal gyrus (BA7) and left precentral gyrus (BA6) (voxel-level *p *< 0.01, cluster-level GRF-corrected *p *< 0.05).

**Table 2.** **Clusters with significant FC increase (peak *t* value) to the dLPFC**.

ROIs	Significant cluster		Peak MNI coordinate			Voxels	Peak *t* value
	AAL atlas	BA atlas	X	Y	Z		
Left dLPFC	Frontal_Sup_Medial_R	BA8_R	6	33	42	119	4.4770
Right dLPFC	Parietal_Inf_L	BA7_L	–30	–78	42	111	3.9540
	Precentral_L	BA6_L	–33	6	45	107	4.3617

Notes: AAL, Anatomical Automatic Labeling; BA, Brodmann Area; dLPFC, dorsolateral prefrontal cortex; FC, functional connectivity; MNI, Montreal Neurological Institute.

**Fig. 1.**
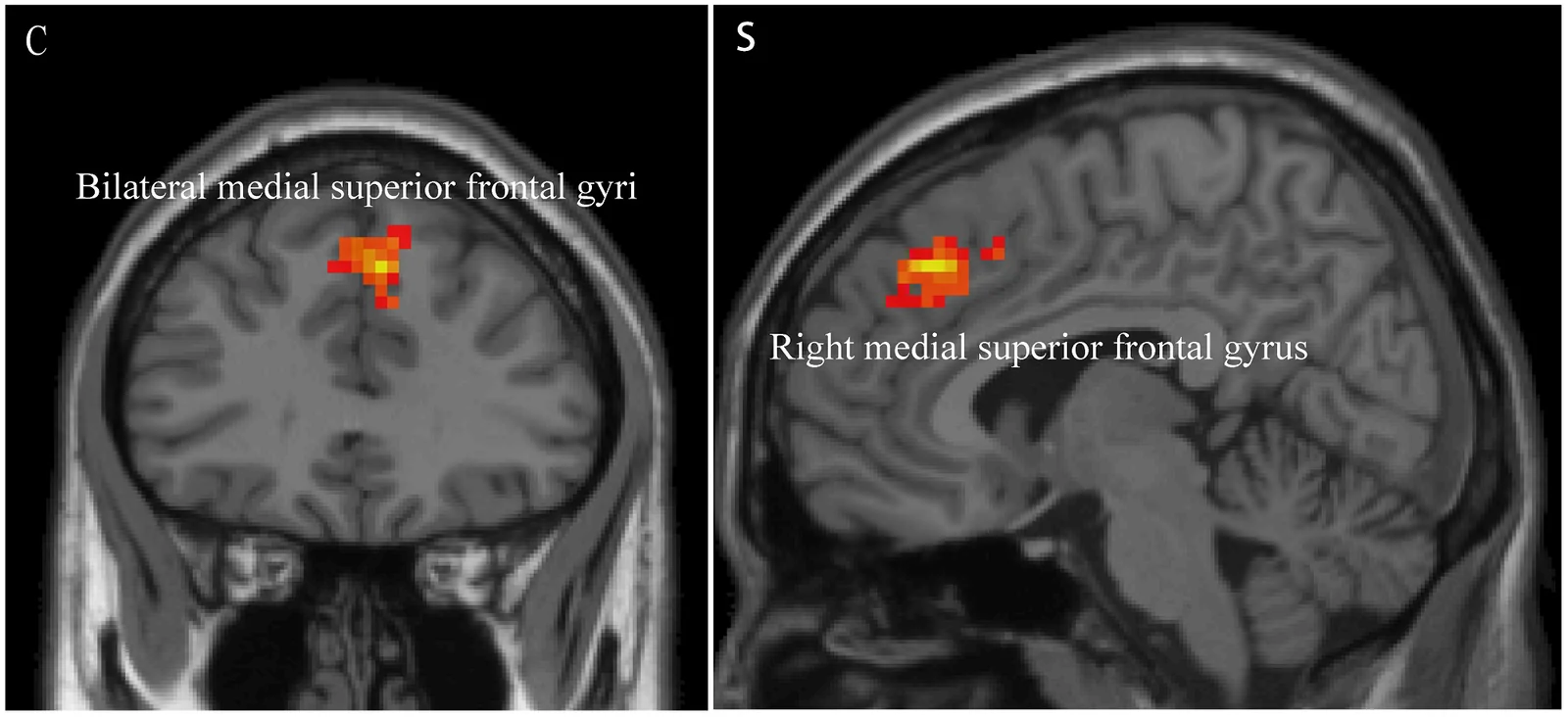
**Clusters with significant FC increase (having more than 30 voxels) to the left dLPFC**. Notes: C, coronal plane; S, sagittal plane.

**Fig. 2.**
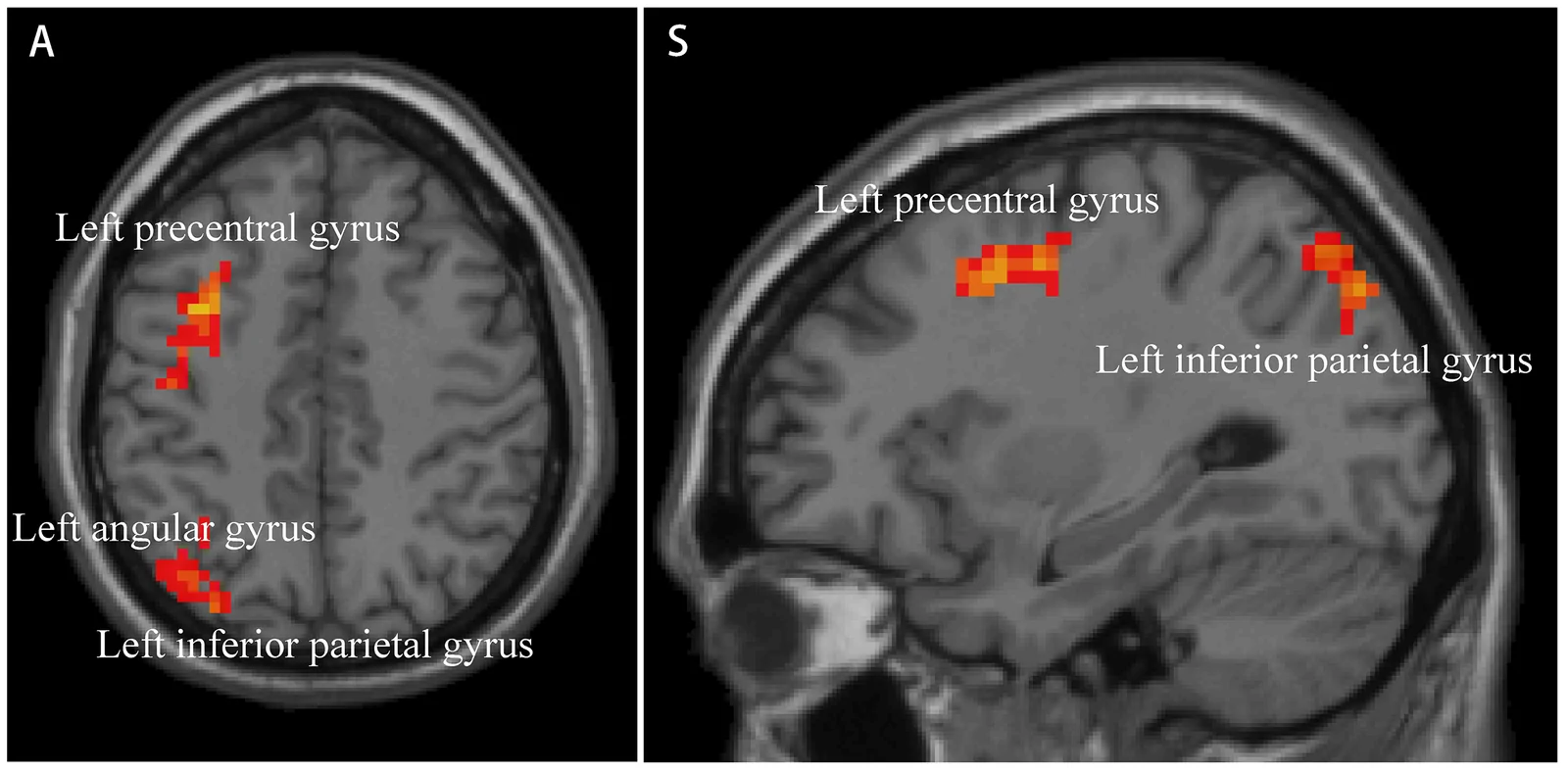
**Clusters with significant FC increase (having more than 30 voxels) to the right dLPFC**. Notes: A, axial plane; S, sagittal plane.

The EC results were summarized in Table 3 and Figs. 3,4,5. With the left dLPFC as the seed, the GAD group demonstrated significantly decreased EC from the left dLPFC to the left caudate nucleus, as well as from the left postcentral gyrus to the left dLPFC. When seeding the right dLPFC, the GAD group showed reduced EC from right dLPFC to left thalamus and right BA37 (voxel-level *p* < 0.01, cluster-level GRF-corrected *p* < 0.05).

**Table 3.** **Clusters with significant EC decrease (peak *t* value) with the dLPFC**.

ROIs	Significant clusters		Peak MNI coordinate			Voxels	Peak *t* value
	AAL atlas	BA atlas	X	Y	Z		
Left dLPFC →	Caudate_L	BA25_L	–15	18	6	168	–4.4801
→ Left dLPFC	Postcentral_L	BA2_L	–45	–30	48	59	–3.8052
Right dLPFC →	Thalamus_L	/	–12	–9	18	224	–4.5574
	/	BA37_R	24	–42	3	78	–4.1983

Notes: EC, effective connectivity.

**Fig. 3.**
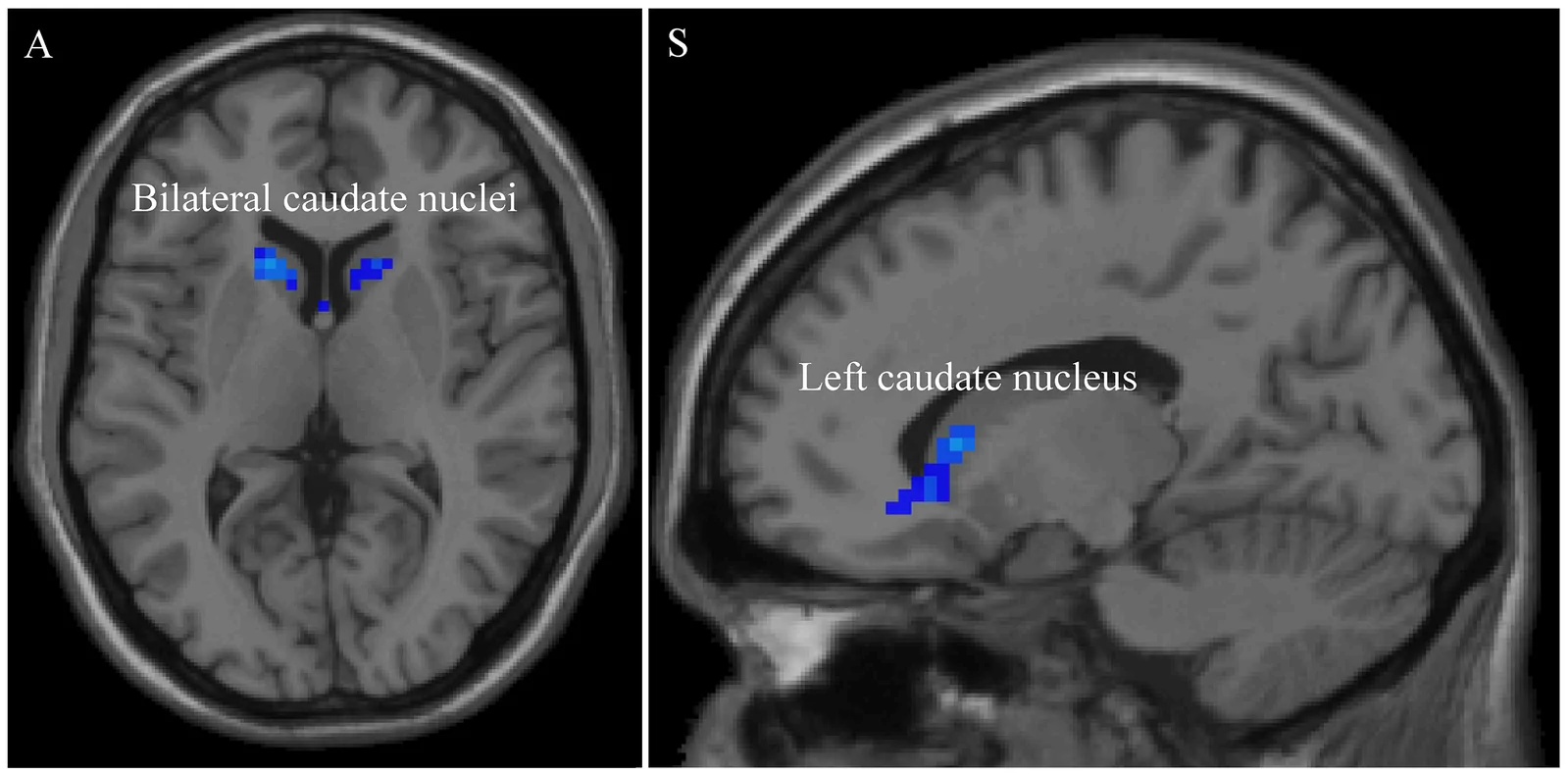
**Clusters with significant EC decrease (having more than 30 voxels) from the left dLPFC**.

**Fig. 4.**
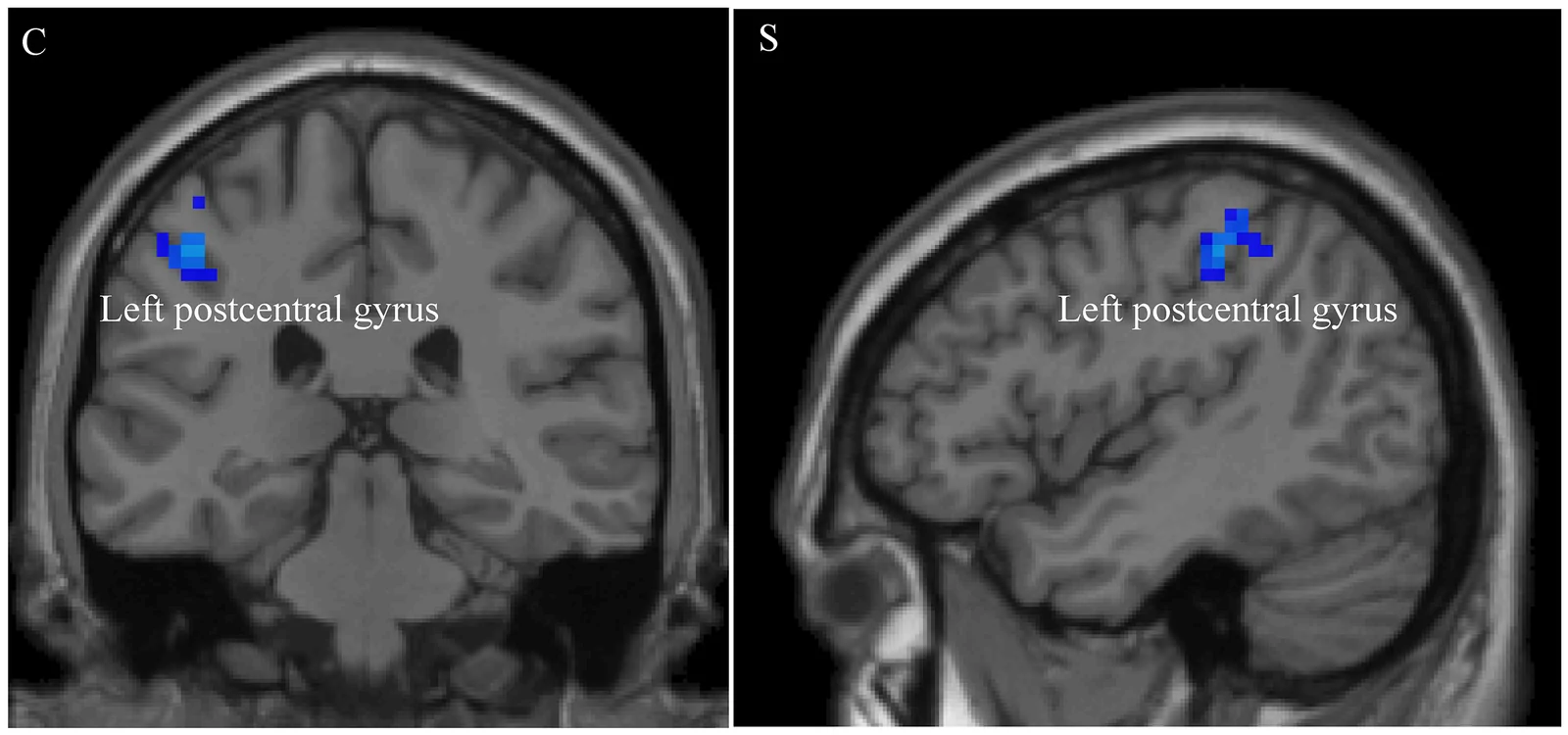
**Clusters with significant EC decrease (having more than 30 voxels) to the left dLPFC**. Notes: C, coronal plane.

**Fig. 5.**
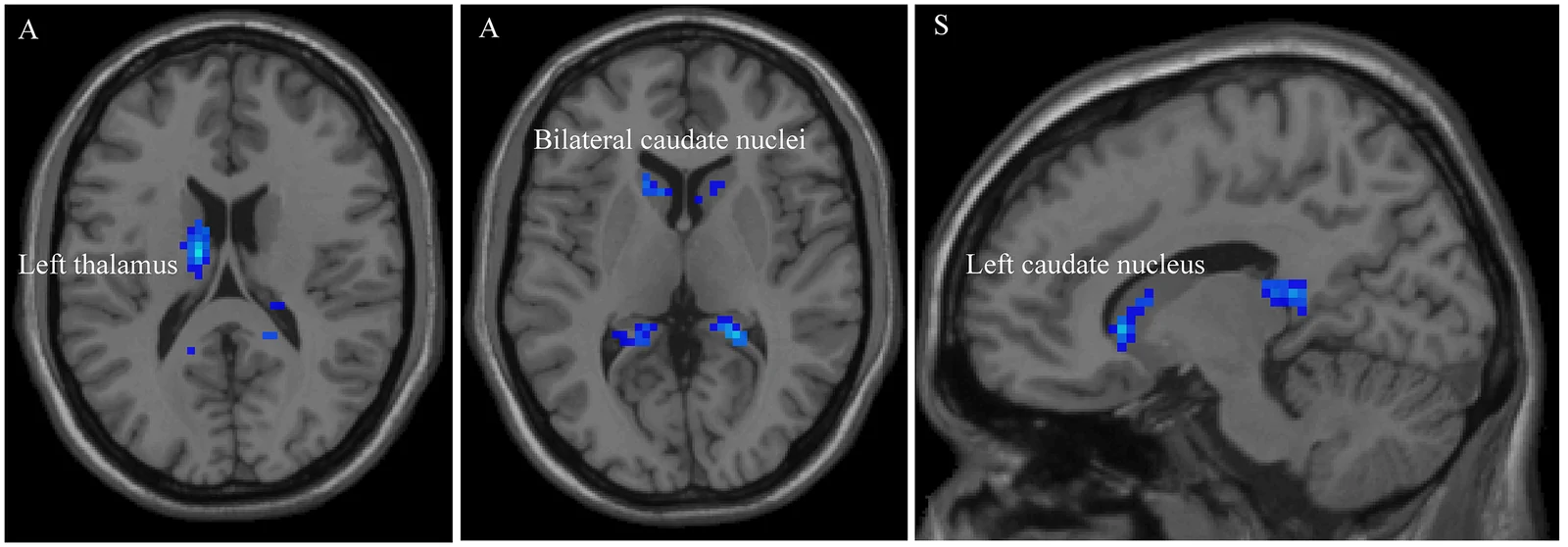
**Clusters with significant EC decrease (having more than 30 voxels) from the right dLPFC**. Notes: A, axial plane; S, Sagittal plane.

### 3.3 Partial Correlation Analysis Between the Strength of rsFC and EC and Clinical Measures of GAD

Given the wide variability in the duration of the current episode, we used it as a covariate in a partial correlation analysis. As shown in Table 4, after controlling for the duration of the current episode, the significantly increased FC between the left dLPFC and right medial superior frontal gyrus was positively correlated with AB scores measured by dot-probe task (r = 0.584, *p* < 0.05) in GAD group. The FC between the right dLPFC and the left precentral showed negative correlations with both AB scores measured by emotional Stroop and GAD-7 (r = –0.515, r = –0.315; *p* < 0.05). Furthermore, the lower EC from the right dLPFC to the left thalamus showed significant negative correlations with AB scores measured by the dot-probe task (r = –0.467, *p* < 0.05). It is important to note that the correlation is no longer significant when we conduct multiple comparison corrections.

**Table 4.** **Partial correlation analysis between functional and effective connectivity values and clinical characteristics in patients with GAD**.

FC/EC value	Clinical measures	Correlation coefficient	*p*
FC between left dLPFC and right medial superior frontal gyrus	AB measured by the dot-probe task	0.584	0.014
FC between right dLPFC and the left precentral	AB measured by the emotional Stroop task	–0.515	0.034
FC between right dLPFC and the left precentral	GAD-7	–0.315	0.046
EC from right dLPFC to left thalamus	AB measured by the dot-probe task	–0.467	0.039

## 4. Discussion

In recent years, research on the pathological mechanisms of GAD has shifted from focusing on regional abnormalities to investigating widespread disruptions in functional brain networks. Building upon previous research findings, this study selected bilateral dLPFC as the seed region to conduct voxel-wise whole-brain analyses of both FC and EC. The results revealed widespread abnormalities in functional and effective connectivity in GAD patients during resting-state, and these aberrant connectivity patterns were significantly correlated with clinical symptom severity, further supporting the central regulatory role of the dLPFC in anxiety-related neural networks. Moreover, our findings provided neuroimaging evidence for the cognitive model of GAD, suggesting that dysregulated connectivity between the dLPFC, limbic system, and sensorimotor networks (SMN) may underlie core symptoms such as AB in GAD patients.

Our findings revealed abnormal connectivity between the dLPFC and core regions of the SMN compared to HCs. The SMN, a core system for sensorimotor integration in the brain, comprises key structures such as the precentral gyrus, postcentral gyrus, supplementary motor area (SMA), and cerebellar lobules IV/V/VI [29]. This network not only integrates tactile, proprioceptive, and motor control functions but also plays a crucial role in complex cognitive decision-making [30]. Dysfunction in the SMN may be associated with altered sensory processing in GAD, such as heightened vigilance toward physiological changes [31].

Existing evidence indicates structural and functional abnormalities in critical SMN regions among GAD patients [32,33,34]. In structural level, meta-analyses have shown larger volumes in the precentral and postcentral gyri but reduced SMA volume in GAD patients [22]. In functional level, GAD patients exhibited hyperactivation in the postcentral gyrus during fear learning and emotion regulation tasks [22]. In connectivity level, studies have reported decreased FC between the dACC and postcentral gyrus, along with increased FC between the superior temporal gyrus and precentral gyrus [34]. A study by Wang et al. [35] also found increased rsFC between the right dLPFC and the precentral gyrus, which is consistent with our findings. Notably, a research found a positive correlation between gray matter volume in the precentral gyrus and HAMA scores, whereas rsFC between the precentral gyrus and superior temporal gyrus was negatively correlated with HAMA scores [34]. Another longitudinal study indicated reduced ALFF and ReHo in the precentral and postcentral gyri of patients with GAD, and hyperactivity in the postcentral gyrus predicted a better response to escitalopram treatment [36]. These studies suggested that the precentral/postcentral gyrus may serve as a potential biomarker for distinguishing GAD patients from HC. From a network interaction perspective, the aberrant connectivity between the dLPFC and SMN regions reflects impaired integration between higher-order cognitive control and primary sensorimotor networks in GAD. On the other hand, our findings of increased FC yet decreased EC between the dLPFC and SMN regions reflect the complexity and heterogeneity of neural network alterations in GAD.

Disrupted inter-network connectivity may underlie the diminished decision-making capacity and avoidance behaviors observed in GAD patients. Correlation analyses revealed significant associations between aberrant FC values and anxiety severity (as measured by GAD-7), and AB indices in GAD patients. These findings suggested that dysconnectivity between dLPFC and SMN may constitute a core pathophysiological mechanism underlying GAD, warranting further investigation.

The executive control network (ECN), composed of the dLPFC, inferior parietal gyrus, and ACC, primarily mediates top-down cognitive regulation [30,37]. This network supports working memory maintenance, attentional processes, and cognitive control coordination [37,38]. Aberrant connectivity within this network may underlie core symptoms of GAD, particularly difficulties in sustained attention. Our study revealed enhanced FC between the dLPFC and IPL, suggesting increased integration within the ECN. Consistent with our findings, a task-based fMRI study investigated neural activation patterns and FC in response to threatening versus neutral stimuli among patients with anxiety disorders (including GAD, PD, and SAD) compared to HCs [39]. The results revealed GAD-specific positive coupling between dLPFC and vlPFC activity [39]. This enhanced FC may reflect GAD patients’ increased cognitive effort to ignore task-irrelevant information and downregulate unpleasant emotions. Notably, Etkin et al. [40] similarly observed amygdala dysfunction co-occurring with compensatory engagement of the ECN. These findings collectively suggest that strengthened connectivity within the ECN may represent enhanced top-down compensatory regulatory activity.

EC analysis revealed significantly reduced directional connectivity from the dLPFC to the key brain regions including the caudate nucleus, thalamus and BA37. As core structures of the limbic system, the thalamus exhibited decreased EC with dLPFC, consistent with previous findings of fronto-limbic network dysfunction, which may reflect impaired top-down regulatory mechanisms of the PFC [22,41,42]. Neuroimaging research has shown that the thalamus is key not only for filtering sensory information, but also for higher cognitive functions and regulating emotions [43,44,45]. Accumulating evidence suggests that weakened FC between the PFC and limbic structures and disrupted integrity of the fronto-limbic network may represent a key neurobiological feature of GAD [45,46]. In this study, GCA provided evidence of altered directional influence from the prefrontal cortex to the limbic system in GAD patients, consistent with the hypothesis of impaired top-down regulation.

Further correlation analyses demonstrate that the strength of dLPFC-thalamus connectivity is negatively correlated with AB toward threatening stimuli. Our findings suggested that impaired regulatory function of the dLPFC over the thalamus may play a critical role in the pathophysiological mechanisms of GAD. However, it should be noted that our correlation analyses were not corrected for multiple comparisons. Given the number of correlations performed, there is an elevated risk of Type I error. However, due to the relatively small sample size, applying a strict correction resulted in no significant findings, and the primary purpose of these analyses was exploratory. Future studies with larger samples are needed to validate these preliminary findings.

The caudate nucleus, as a crucial component of the striatum, forms the basal ganglia network system together with the basal ganglia, substantia nigra, subthalamic nucleus, global pallidum externa and interna [38]. Current research indicated that this network plays a pivotal role in regulating goal-directed behaviors including emotion, motivation, and cognition [47]. In adolescents with SAD, researchers have observed hypersensitivity of the caudate nucleus to stimuli [48]. Moreover, patients with GAD exhibit significantly reduced regional homogeneity (ReHo) in the left caudate nucleus, along with weakened FC between the caudate nucleus and amygdala [48]. Pharmacological studies have found that citalopram reduced activation in striatum [49]. Of particular note, fMRI studies in individuals with trait anxiety have revealed important neural mechanisms: during sustained attention to response Task (SART), these individuals show weakened FC between the right dLPFC and bilateral caudate nucleus and thalamus [50]. Researchers hypothesized that sustained attentional control may rely on the recurrent activation of the lateral prefrontal regions and the frontal-thalamo-striatal circuit, and that reduced connectivity in this circuit is closely associated with attentional control deficits in trait anxiety [50]. Our findings provide empirical support for this theoretical model.

This study also found a decrease in the EC from the dLPFC to the right BA37. The brain region mainly corresponding to BA37 is the fusiform gyrus, which is part of the temporal lobe and occipital lobe that has been suggested to play an important role in face, body, and word recognition [51]. Abnormal activities in the fusiform gyrus in response to emotional faces have been reported in different anxiety disorders [52,53,54]. A study found that patients with GAD exhibited increased FC between the hippocampus and fusiform gyrus, with the connection strength correlating with symptom severity [55]. The authors suggested that the fusiform gyrus-hippocampus connectivity may serve as a biomarker for GAD severity [55]. In this study, the reduced EC from the dLPFC to the fusiform gyrus reflects diminished regulatory control of emotional regulation brain regions over sensory processing areas.

Taken together, these findings are best understood at the network level rather than as isolated regional abnormalities. They suggest a disrupted integration between the ECN and sensorimotor/subcortical systems. Abnormal connectivity of the dLPFC with the right BA37, the thalamus and the caudate nucleus points to a dysregulated threat processing circuit. Thus, our results do not merely indicate focal dLPFC dysfunction, but may also reflect more widespread network abnormalities.

Beyond the traditional view of prefrontal top-down control, emerging frameworks emphasize that dLPFC function is tightly linked to cortical excitability and the adaptive regulation of neural gain, which may translate into individual differences in behavioral flexibility under affective challenge [56]. Within this perspective, the altered directional connectivity observed in our GAD sample may reflect a suboptimal tuning of prefrontal excitability, whereby reduced causal outflow toward sensorimotor and subcortical targets compromises the adaptive recalibration of attentional and motor responses to threat-related cues. This view offers a complementary mechanistic account linking our connectivity findings to the behavioral Stroop interference observed in the same patients.

An apparent discrepancy in our results deserves further consideration. Although the group-level comparison revealed significantly elevated AB scores in the emotional Stroop task but not in the dot-probe task, correlation analyses nonetheless identified meaningful associations between FC/EC values and dot-probe bias scores. This pattern may reflect the fact that the two paradigms capture different components of attentional bias: the emotional Stroop task primarily indexes sustained attentional interference from threat-related content, whereas the dot-probe task reflects earlier, automatic orienting of attention [57]. Furthermore, the absence of a group-level difference does not preclude clinically meaningful individual variability within the patient group, which may still correlate with underlying neural alterations. The convergence of behavioral and neural findings across different attentional components strengthens the inference that dLPFC-related connectivity underlies multiple facets of threat-related attentional processing in GAD.

In summary, this study identified an interesting pattern: compared with HC, FC between the dLPFC and several regions (such as sensorimotor and subcortical areas) was increased, while EC was decreased. This apparent dissociation may be interpreted through several non-exclusive frameworks. First, from a compensatory perspective, the enhanced undirected FC may reflect a maladaptive compensatory response to the weakened top-down causal modulation from the dLPFC. The EC from the dLPFC to emotion-related brain regions is reduced, indicating a decline in its top-down regulatory efficacy. To maintain behavioral performance or basic neural communication, the system has to “recruit” additional neural resources, manifesting as abnormally enhanced synchrony (FC) with these regions. Second, from a methodological standpoint, FC captures temporal co-fluctuations regardless of directionality, whereas GCA quantifies directional predictability; the two measures may therefore reveal complementary rather than redundant aspects of network organization. Third, from a neurobiological perspective, hyperconnectivity in undirected metrics alongside diminished directional influence may represent a hallmark of network dysregulation in chronic anxiety states.

It is noteworthy that our whole-brain voxel-wise analyses did not reveal significant FC or EC alterations involving the amygdala, despite the prominence of the amygdala in classical prefrontal–limbic models of anxiety [58]. Several factors may account for this observation. First, the amygdala is susceptible to BOLD signal dropout due to its proximity to air–tissue interfaces, which may reduce sensitivity in whole-brain analyses. Second, our sample consisted of medication-naïve patients with mild-to-moderate severity, which may differ from more severe or medicated cohorts in which amygdala abnormalities are typically reported. Third, the absence of amygdala findings, together with the presence of sensorimotor and basal ganglia abnormalities, may suggest that dLPFC-related dysregulation in GAD extends beyond the classical fronto-amygdala circuit—a possibility that warrants further investigation with targeted ROI-based and task-based designs.

### Limitations

When interpreting the findings of this study, the following limitations should be considered. First, this study employed a cross-sectional design, failing to capture the dynamic changes in FC/EC across the acute and remission phases of the illness. Future longitudinal studies may be better suited to elucidate the characteristics of connectivity alterations and determine whether these changes represent trait markers or state markers of GAD. Second, the participants were predominantly medication-naïve individuals with mild-to-moderate GAD, and the HAMA scores exhibited a relatively narrow range. This may limit the generalizability of the results. Third, Our EC analysis was based on resting-state GCA, our results reflect the ‘directional predictability’ of the BOLD signal, rather than direct ‘causality’. Finally, the relatively small sample size somewhat diminished the statistical power.

## 5. Conclusions

In summary, this study found that, compared to HCs, patients with GAD exhibited abnormalities in both FC and EC based on the bilateral dLPFC. This suggested dysfunction in core brain regions involved in emotional regulation and cognitive function. These findings further confirm the central role of the dLPFC in the pathological mechanisms of GAD. Beyond abnormalities in fronto-limbic network connectivity, GAD also demonstrates aberrant connectivity patterns in brain regions associated with the sensorimotor network and the basal ganglia network, thereby enriching the neuropathological model of GAD. Notably, the abnormal connectivity between the dLPFC and the sensorimotor regions and thalamus was closely associated with AB. This indicates that dLPFC-related abnormalities in functional and effective connectivity may play a significant role in the selective attention to threat-related stimuli observed in GAD patients. Therefore, neuromodulatory interventions targeting the dLPFC may represent a potential therapeutic target, warranting further investigation in controlled clinical trials. Furthermore, our results were obtained from patients without comorbidities and who were medication-naive, effectively excluding the potential confounding effects of comorbidities and medications.

## Data Availability

Due to the clinically sensitive nature of the research and the conditions of our ethics approval, supporting data cannot be made openly available.
